# Rheology of Naturally Deformed Antigorite Serpentinite: Strain and Strain‐Rate Dependence at Mantle‐Wedge Conditions

**DOI:** 10.1029/2022GL098945

**Published:** 2022-08-26

**Authors:** C. J. Tulley, Å. Fagereng, K. Ujiie, S. Piazolo, M. S. Tarling, Y. Mori

**Affiliations:** ^1^ School of Earth and Environmental Sciences Cardiff University Cardiff UK; ^2^ Faculty of Life and Environmental Sciences University of Tsukuba Tsukuba Japan; ^3^ School of Earth and Environment University of Leeds Leeds UK; ^4^ Department of Geology University of Otago Dunedin New Zealand; ^5^ Now at Department of Earth and Planetary Sciences McGill University Montreal QC Canada; ^6^ Kitakyushu Museum of Natural History and Human History Kitakyushu Japan

**Keywords:** antigorite serpentine, plate boundary, deformation mechanisms, rheology

## Abstract

Antigorite serpentinite is expected to occur in parts of subduction plate boundaries, and may suppress earthquake slip, but the dominant deformation mechanisms and resultant rheology of antigorite are unclear. An exhumed plate boundary shear zone exposed near Nagasaki, Japan, contains antigorite deformed at 474°C ± 30°C. Observations indicate that a foliation defined by (001) crystal facets developed during plate‐boundary shear. Microstructures indicating grain‐scale dissolution at high‐stress interfaces and precipitation in low‐stress regions suggest that dissolution‐precipitation creep contributed to foliation development. Analysis of crystal orientations indicate a small contribution from dislocation activity. We suggest a frictional‐viscous rheology for antigorite, where dissolution‐precipitation produces a foliation defined by (001) crystal facets and acts to resolve strain incompatibilities, allowing for efficient face‐to‐face sliding between facets. This rheology can not only explain aseismic behavior at ambient plate boundary conditions, but also some of the contrasting behaviors shown by previous field and laboratory studies.

## Key Points

- Outcrops of deformed antigorite serpentinite provide a chance to constrain deformation mechanisms at natural shear zone conditions
- Microstructural observations indicate that the dominant deformation mechanisms are dissolution‐precipitation creep and frictional sliding
- Development of a fine‐grained and foliated fabric with progressive strain leads to reduced effective viscosity

## 1 Introduction

Thermomechanical models of subduction zones require substantial decoupling along the plate boundary in order to produce natural subduction zone geometries (Gerya et al., 2008) and fit heat flow observations (e.g., Abers et al., 2006; Wada et al., 2008). Antigorite serpentine forms in hydrous ultramafic rocks at temperatures ∼300°C–650°C and pressures up to ∼6 GPa (Evans, 2004; Ulmer & Trommsdorff, 1995), and is inferred to occur in the mantle wedge (Reynard, 2013). Serpentinites are often described as having better‐developed ductile deformation structures in comparison to adjacent rocks, suggesting mechanical weakness (Hoogerduijn Strating & Vissers, 1991; Tarling et al., 2019). Accordingly, it has been inferred that serpentinite contributes to decoupling and aseismic behavior (e.g., Reynard, 2013; van Keken, 2003; Wada et al., 2008). However, the mechanisms controlling the strength and rheology of antigorite are unclear (Amiguet et al., 2014; Auzende et al., 2015), and stronger, velocity‐weakening behavior at higher strain rates may allow earthquake nucleation in antigorite (Proctor et al., 2014; Wang et al., 2020).

Laboratory experiments at 300°C–650°C, confining pressures of a few GPa, and dry conditions (except for French et al., 2019) have produced a range of antigorite microstructures and mechanical responses, reflecting a variety of deformation mechanisms. Crystal distortions indicative of dislocation‐related deformation are commonly observed (Amiguet et al., 2014; Auzende et al., 2015; Burdette & Hirth, 2022; Chernak & Hirth, 2010; French et al., 2019; Hilairet et al., 2007; Hirauchi et al., 2020; Proctor & Hirth, 2016; Shao et al., 2021). However, except for in low‐strain experiments by Hilairet et al. (2007), and constant‐stress (rather than strain rate) experiments by Burdette and Hirth (2022), dislocation‐related microstructures are crosscut by localized shear zones that show cataclastic textures, indicating frictional deformation. Many experiments demonstrate that strength depends on strain rate (Chernak & Hirth, 2010; French et al., 2019; Hilairet et al., 2007; Proctor & Hirth, 2016; Shao et al., 2021), consistent with a viscous deformation mechanism. However, strengths dependent on confining pressure are also observed, with dependence increasing toward lower temperature (Chernak & Hirth, 2010; Hirauchi et al., 2020; Proctor & Hirth, 2016; Raleigh & Paterson, 1965; Shao et al., 2021), indicative of frictional behavior. On the basis of these microstructures and mechanical responses, antigorite is commonly interpreted to have a frictional‐viscous rheology controlled by dislocation glide and a frictional mechanism (e.g., Chernak & Hirth, 2010).

Structures within natural antigorite shear zones such as those exposed in the Voltri massif, Italy (300°C–640°C, 0.6–2.2 GPa; Auzende et al., 2015; Hermann et al., 2000), Zermatt‐Saas zone, Western Alps (550°C ± 50°C, 2 ± 0.5 GPa; Wassmann et al., 2011), Sanbagawa belt, Japan (465°C ± 15°C, 1.01 ± 0.06 GPa; Hirauchi et al., 2021), Cerro del Almirez massif, Spain (615°C ± 15°C, 1.75 ± 0.15 GPa; Padrón‐Navarta et al., 2012) and Val Malenco, central Alps (Liu et al., 2020) provide a chance to constrain deformation mechanisms at natural strain rates. Observations of crystal distortion (Auzende et al., 2015; Hirauchi et al., 2021; Padrón‐Navarta et al., 2012) suggest dislocation‐related deformation, which some interpret as the dominant deformation mechanism (Hirauchi et al., 2021; Padrón‐Navarta et al., 2012). Antigorite formed in strain shadows (Hirauchi et al., 2021; Wassmann et al., 2011), and microfractures (Auzende et al., 2006, 2015) indicates local precipitation, and truncated chemical zoning patterns suggest local dissolution (Liu et al., 2020). Such observations are consistent with dissolution‐precipitation creep, which is interpreted by Wassmann et al. (2011) to be the dominant deformation mechanism.

The Nishisonogi metamorphic rocks (NMR) exposed near Nagasaki, Japan contain subducted metasediments, metabasalts and antigorite serpentinite, and are inferred to have hosted subduction plate boundary deformation at 440°C–524°C (Mori et al., 2019; Nishiyama, 1989). We show that antigorite rheology involves components of dislocation creep, fracturing and frictional sliding, and dissolution‐precipitation creep. However, we demonstrate that the dominant mechanisms are dissolution‐precipitation creep and frictional sliding. These mechanisms drive the formation of a mechanically weak foliation defined by aligned (001) facets, resulting in progressive, strain‐dependent weakening.

## 2 Methods

**Figure 1:**
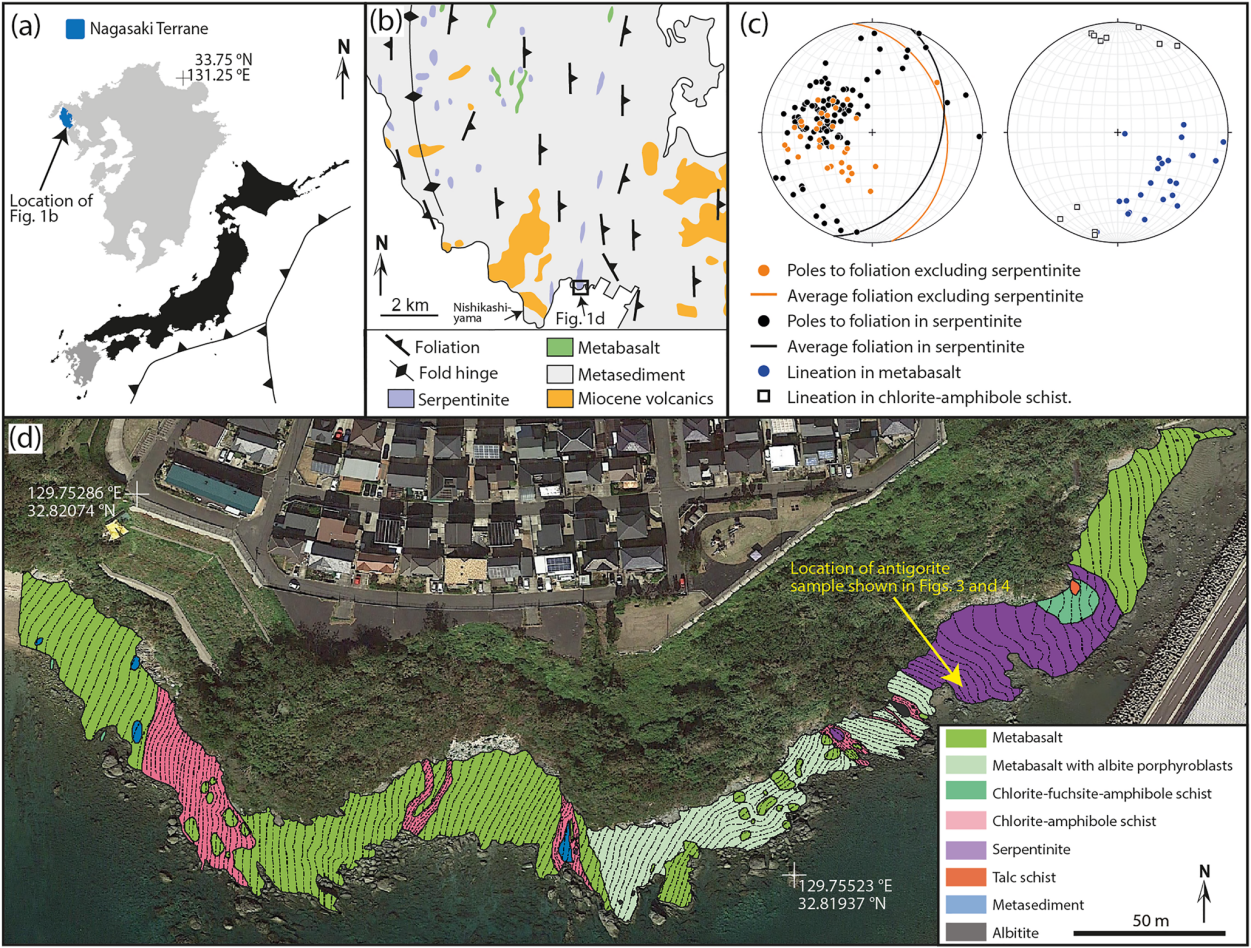
Location and geological maps of the Nishisonogi metamorphic rocks (NMR) at Mie. (a) Location of the study area. (b) Geological map of the NMR on Nishisonogi Peninsula, modified from Nishiyama (1989). (c) Equal area, lower hemisphere stereoplots showing fabric orientations for the area in (d). (d) Geological map of the NMR at Mie (coordinates ‐ WGS84; Satellite imagery ‐ Google; Image ‐ Landsat/Copernicus). Dashed lines indicate the trace of foliation surfaces.

We examined outcrop‐scale structures within serpentinite exposed at Mie, near Nagasaki, Japan (Figure 1a). Polished thin sections for microstructural analysis were prepared in the *x*‐*z* plane of the finite strain ellipsoid. Electron images were produced using a Zeiss Sigma Field Emission Gun Scanning Electron Microscope (FEG‐SEM) at the School of Earth and Environmental Sciences, Cardiff University. EBSD data were collected using an Oxford Instruments Symmetry EBSD detector in an FEI Quanta FEG‐SEM at the School of Earth and Environment, University of Leeds. EBSD patterns were collected at a step size 0.1 μm using a 20 kV electron beam. The Oxford Instruments program AZtec Crystal and the mtex toolbox for matlab (Bachmann et al., 2010) were used to process the data. A description of the processing routine is provided with the EBSD data. Raman spectra were collected using a WITec Alpha 300R + confocal Raman microscope in the Department of Chemistry, University of Otago, New Zealand. Analytical and data processing methods followed those described in Rooney et al. (2018).

## 3 Geological Setting and Outcrop Observations

**Figure 2:**
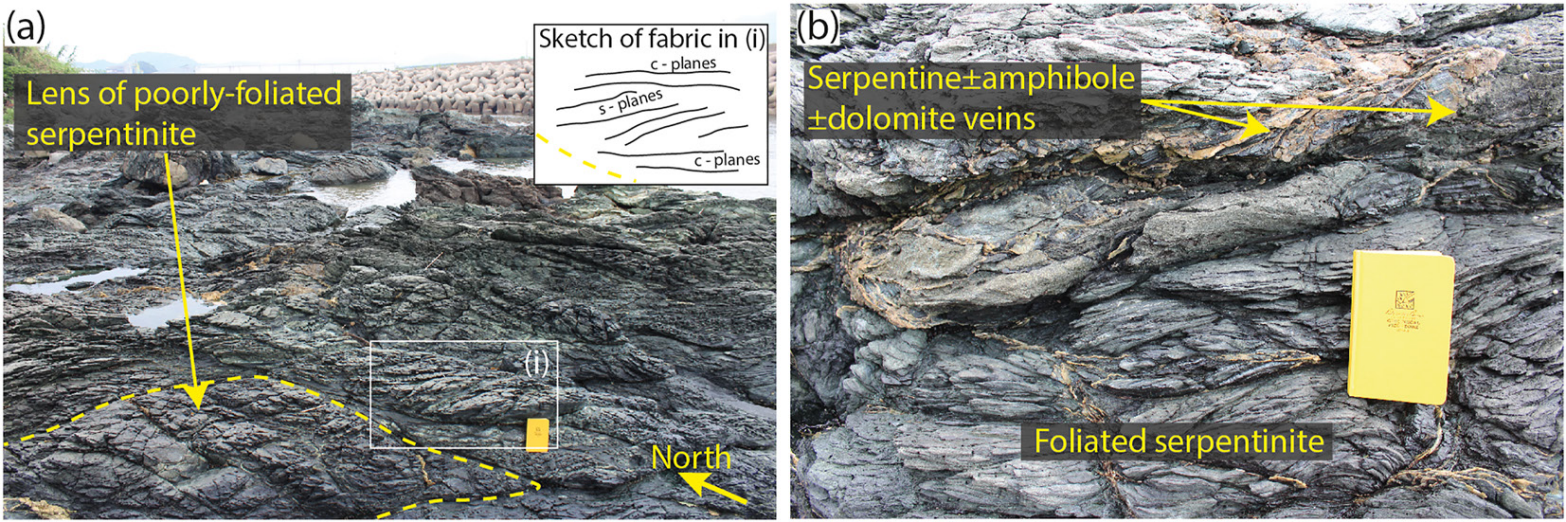
Typical structures in serpentinite outcrops. (a) Foliated serpentinite showing S/C foliation geometry consistent with top‐south shear, enclosing a lens (outlined by the yellow dashed line) of less intensely foliated serpentinite. Photograph looks north‐east from the sample location shown in Figure 1d. (b) Serpentine ± amphibole ± dolomite veins crosscutting and aligned to serpentinite foliation.

The NMR occur within the Nagasaki Terrane which has a Late Cretaceous metamorphic age (Nishiyama, 1989; Wallis et al., 2020). Outcrops at Mie show a layer of serpentinite with a foliation‐normal thickness ∼130 m within schistose, intercalated metabasalt and metasediment (Figures 1b and 1d). The peak temperature experienced by foliation‐defining carbonaceous material in NMR metasediment near Mie is 474°C ± 30°C (Mori et al., 2019), consistent with thermodynamic modeling of a metabasalt mineral assemblage (Tulley et al., 2022). The foliation is continuous between metasediment, metabasalt and serpentinite units (Figures 1b and 1d) and generally dips gently to the east (Figure 1c), although it is folded near lithological contacts (Figure 1d). The lineation plunges gently to the south‐east, except in layers of chlorite‐amphibole schist (intensely foliated metabasalt) that show north‐south trending lineation. S/C foliation geometry in serpentinite (Figure 2a) and chlorite‐amphibole schist (Tulley et al., 2020) indicate top‐south shear sense. In places, S/C foliation geometry in metabasalt indicates top‐north shear, however, this is less common than top‐south S/C geometries, and occurs near competent blocks within the shear zone, suggesting that these structures formed during rotation of competent blocks during bulk top‐south shear.

Within the serpentinite unit, competent meter‐scale lenses of poorly foliated serpentinite occur within a more intensely foliated serpentinite matrix that shows a scaly fabric (Figure 2a). The scaly fabric is defined by foliation anastomosing around competent cm‐scale lenses of poorly foliated serpentinite. The term competency is used to indicate a relative viscosity based on outcrop and microstructural observations. Veins of serpentine ± amphibole ± dolomite occur within the serpentinite. Some veins cross‐cut the foliation and others appear stretched subparallel to the foliation (Figure 2b).

## 4 Microstructure of Serpentinite

**Figure 3:**
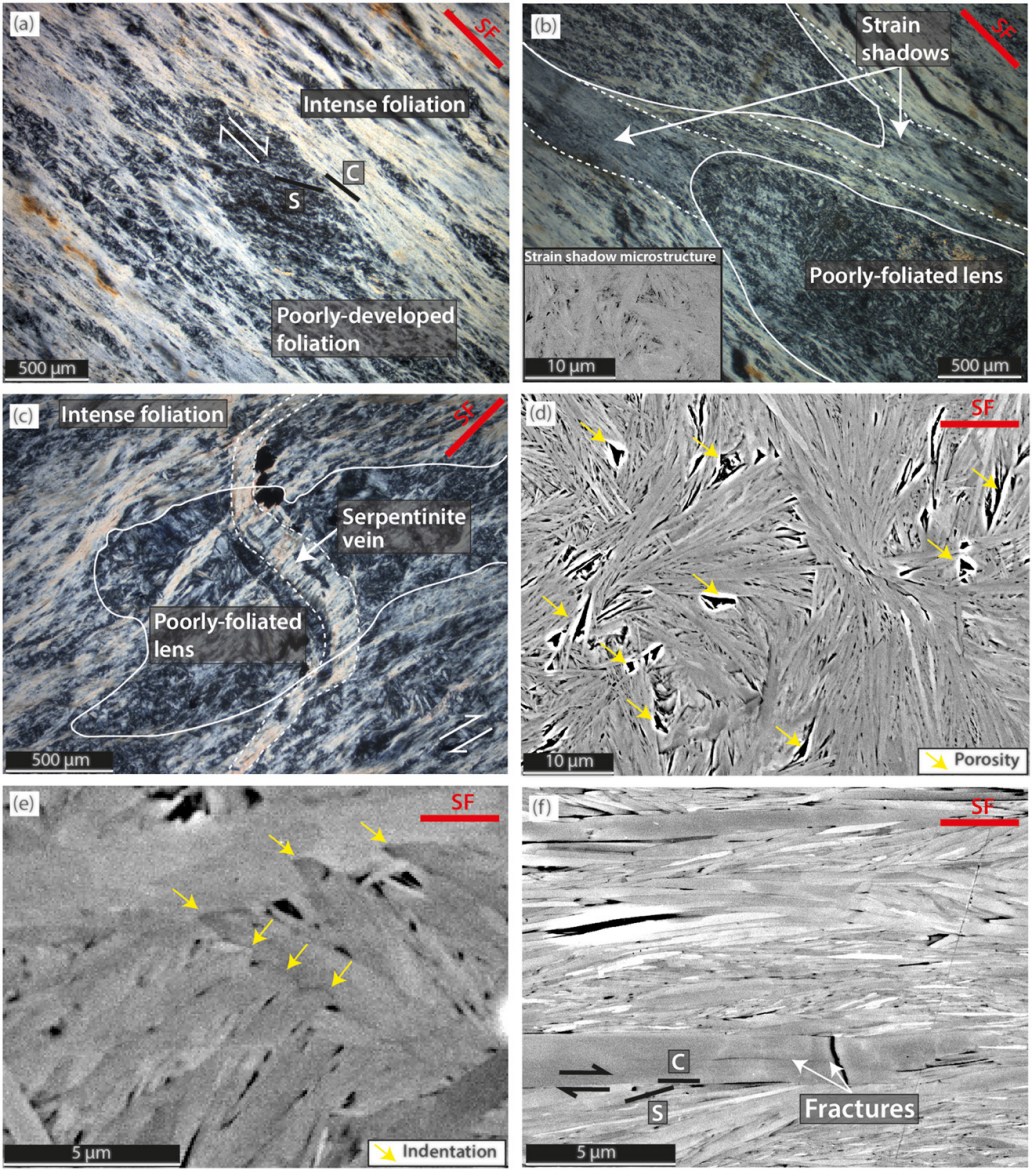
Cross‐polarized light photomicrographs (a–c) and back‐scattered electron images (d–f) showing serpentinite microstructures. Red line “SF” indicates the orientation of the sample foliation, and opposing arrows indicate inferred shear sense. (a) Competent lens of poorly foliated serpentinite within a more intensely foliated matrix. (b) Fine‐grained poorly foliated serpentine in strain shadows adjacent to competent lenses. (c) Serpentine vein cross‐cutting a poorly foliated lens and deflected along foliation planes within a more intensely foliated matrix. (d) Typical interpenetrating microstructure of poorly foliated serpentinite. (e) Indentation structures along grain boundaries. (f) Typical microstructure of intensely foliated serpentinite.

The dominant microstructure is a foliation defined by (001) antigorite facets. The foliation wraps around competent lenses of less intensely foliated serpentinite, and shows S/C geometry (Figures 3a and 3b); similar to the structure observed at the outcrop‐scale (Figure 2a). Fine‐grained poorly foliated serpentinite forms strain shadows adjacent to some competent lenses (Figure 3b). Spatial changes in the intensity of the foliation are generally gradual. Raman spectra of serpentine show the characteristic antigorite peaks near 3,663 and 3,695 cm^−1^ (e.g., Petriglieri et al., 2015; Rooney et al., 2018). Overall, Raman spectra suggest the polytype is antigorite, consistent with EBSD pattern indexing, and imply that there is no variation in polytype within the sample. A detailed interpretation of the Raman spectra is given in Supporting Information S1.

In the least intensely foliated serpentinite, grains are generally <1 μm wide and <50 μm long and form an interpenetrating fabric with no preferred grain orientation (Figure 3d). This interpenetrating fabric is common in antigorite serpentinite (e.g., Hirauchi et al., 2010; Rouméjon et al., 2019; Vogler, 1987; Wicks & Whittaker, 1977; Williams, 1979). In regions with a moderately developed foliation, grain long‐axes which lie close to (001) facets are preferably aligned with the bulk foliation. Additionally, notches are observed in grain boundaries (Figure 3e). These notches are most prominent where (001) facets intersect at high angles. In the most intensely foliated serpentinite, most (001) facets are closely aligned (Figure 3f), and misalignments commonly define S/C geometries (Figure 3f). Grains are typically <0.5 μm wide and <20 μm long; finer than in poorly foliated regions where the length of short axes is < 1 μm. Porosity appears to decrease with increasing foliation intensity (Figures 3d and 3f). Where serpentine veins crosscut the foliation they are thinned and deflected toward the foliation in more intensely foliated regions (Figure 3c).

**Figure 4:**
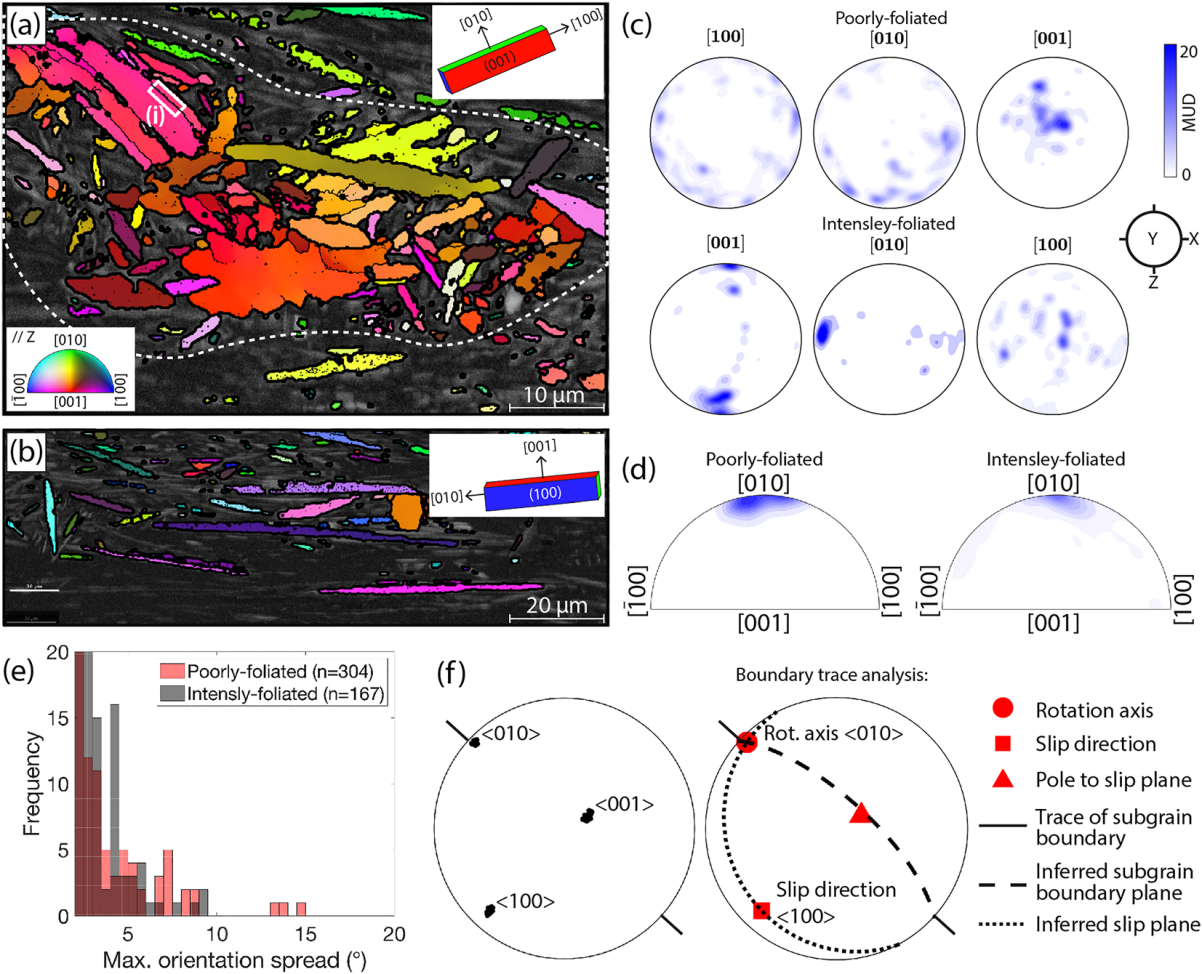
EBSD data showing differences between poorly and intensely foliated serpentinite; (a) EBSD band contrast map overlain by colors corresponding to crystal orientation relative to the page normal. Thick and thin black lines indicate the boundaries of grains and subgrains, respectivly. The yellow dashed polygon outlines a poorly foliated region from which data shown in plots (c–e) were sourced. Region (i) shows the subgrain boundary analyzed in (f). The inset in the upper right shows a typical orientation of the antigorite unit cell. (b) As in (a), but for an intensely foliated region of the sample. Note that if dislocation glide is assumed to be the dominant deformation mechanism, grain orientations suggest that the dominant slip system was <010> (001). (c) Lower‐hemisphere, equal area pole figures of orientation measurements. Contours show multiples of a uniform distribution (MUD). (d) Pole figures in crystal coordinates showing the distribution of misorientation axes for subgrain boundaries, with contours as in (c). (e) Histograms showing maximum difference between an orientation measurement and the grain mean orientation. (f) Pole figures showing the orientation of crystallographic axes across a subgrain boundary (left), and the related boundary trace analysis (right) which shows that data is consistent with a tilt boundary formed by edge dislocations related to the slip system <100> (001).

Poorly foliated antigorite shows numerous subgrain boundaries (defined as boundaries where misorientation is 2°–10°) (Figure 4a) and a preferred [001] orientation perpendicular to foliation (Figure 4c). Intensely foliated antigorite shows fewer subgrain boundaries (Figure 4b), and smaller degrees of internal distortion (Figure 4e), but a more intense crystallographic preferred orientation (CPO) with [001] normal to foliation [010], parallel to lineation and [100] within the foliation plane, perpendicular to lineation (Figure 4c). In both poorly and intensely foliated serpentinite, subgrain boundary misorientation axes are concentrated near [010] (Figure 4d). Pole figures of the main crystal axes adjacent to subgrain boundaries typically show [100] and [001] in small‐circle distributions, and little dispersion around [010] (Figure 4f).

## 5 Development of the Serpentinite Microstructures

Interpenetrating microstructure similar to that observed in poorly foliated antigorite from Mie has previously been interpreted to develop in low‐strain environments (Maltman, 1978; Morales et al., 2018). Thermomechanical models (e.g., Abers et al., 2006; Syracuse et al., 2010; Wada et al., 2008) indicate that a shallow portion of the mantle wedge is decoupled from the subducting slab, and may be a low‐strain environment (Wada et al., 2008) where an interpenetrating fabric could develop. S/C foliation geometry (Figure 2a) implies top‐south shear, consistent with north‐trending subduction along the eastern Eurasian margin in the Late Cretaceous (Whittaker et al., 2007). As foliation is continuous between metasediment, metabasalt and serpentinite (Figures 1b and 1d), we infer that the serpentinite foliation developed at 474°C ± 30°C, as inferred for metasediment (Mori et al., 2019) and metabasalt (Tulley et al., 2022) foliations in the Mie area.

**Figure 5:**
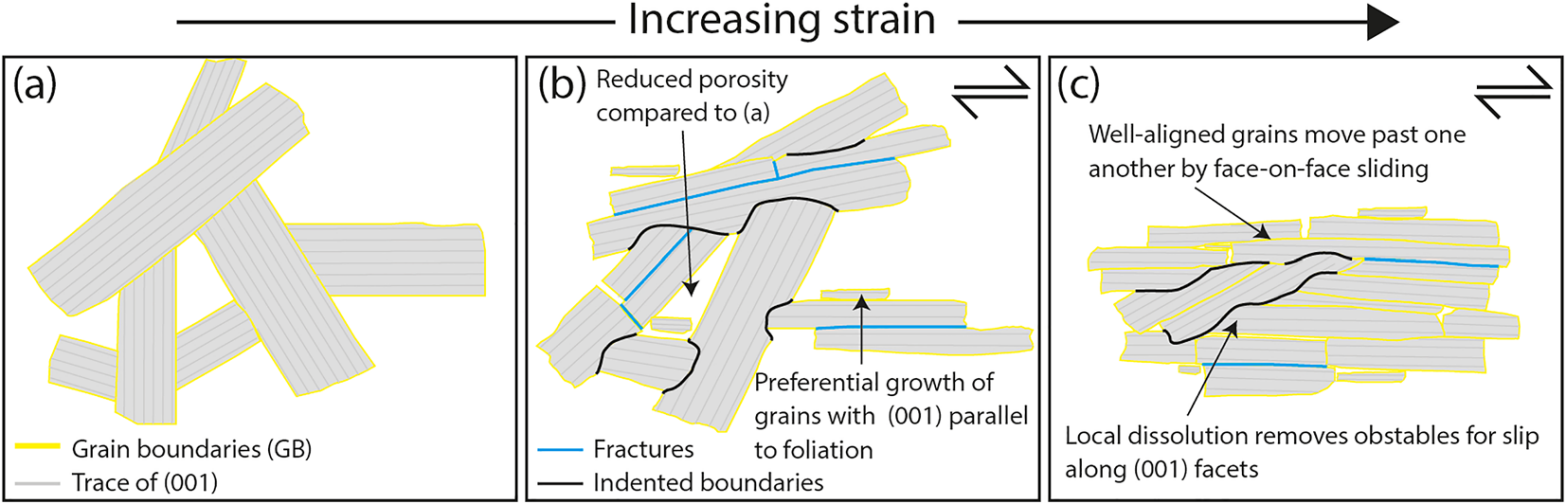
Sketches showing the process of fabric development. (a) Pre‐deformation; grains form an interpenetrating fabric. (b) Grains rotate toward the local shear plane and sliding occurs between (001) facets. Strain incompatibilities are resolved dominantly by dissolution‐precipitation creep. (c) Increased strain results an increasingly intense foliation defined by aligned (001) facets.

The interpenetrating microstructure of poorly foliated antigorite (Figure 3d) should inhibit frictional slip or dislocation glide, as misaligned adjacent grains represent geometrical asperities for shear along mechanically weak (001) surfaces (Figure 5; Hansen et al., 2020). Deformation that is required to remove geometrical asperities and accommodate bulk strain is controlled by the most efficient of three main mechanisms, each favored at different environmental conditions (e.g., Knipe, 1989; Rutter, 1976); (a) fracturing and frictional sliding, (b) the generation and movement of dislocations, and (c) diffusive mechanisms such as dissolution‐precipitation creep.

The presence of subgrain boundaries (Figures 4a and 4b), which are a signature of dislocation activity (Drury & Urai, 1990), and the preferred orientation of misorientation axes (Figure 4d) suggests that some deformation occurred by dislocation activity. For a region along a typical subgrain boundary (Figure 4a) the orientation of crystal axes are shown in Figure 4f. The axis that shows the least dispersion is [010], consistent with the statistically prominent misorientation axis shown in Figure 4d. If the boundary is a tilt wall, the boundary plane must contain the rotation axis and the boundary trace. Alternatively, for a twist wall the misorientation axis must be normal to the boundary plane. In the example shown in Figure 4f, only a tilt boundary with a rotation axis of <010> is consistent with the data, hence the boundary is interpreted as a tilt wall. As the dislocation slip direction must be 90° from the rotation axis and not within the boundary plane, the slip direction must be <100>, implying the dominant slip system is <100> (001) (Figure 4f, right).

On the other hand, the CPO of antigorite in well‐foliated regions shows [010] subparallel to lineation. This contrasts with the results of the boundary trace analysis (Figure 4f, right), because the crystal axis subparallel to lineation should be the slip direction if dislocation activity is a dominant deformation mechanism. Therefore, because the observed CPO is inconsistent with the geometries of subgrain boundaries (Figures 4d and 4f, right), dislocation activity cannot have been the dominant mechanism responsible for the formation of the foliation. While we draw this conclusion for the Mie example, the effectiveness of antigorite dislocation creep in general is fundamentally restricted by that all observed slip systems are subparallel to the basal plane, thereby restricting the geometry of dislocation‐related strains (Figure 4d; Chernak & Hirth, 2010).

Antigorite formed in strain shadows around mm‐scale competent lenses (Figure 3b) and antigorite veins that cross‐cut foliation but are also deflected parallel to foliation (Figure 3c), imply precipitation of antigorite grains during the foliation‐forming deformation. As mineral grains grow fastest parallel to the direction of least compressibility (Kamb, 1959) and antigorite compressibility is substantially lower in directions normal to [001] than in other crystallographic directions (Bezacier et al., 2010), growth of (001) facets is thermodynamically favored. Because grain growth in a stressed system will result in the fastest growing direction being aligned with the lineation, such anisotropic grain growth should assist foliation development (Figure 5), and may explain observations of reduced porosity with increasing foliation intensity (Figures 3d and 3f).

To explain observations of distributed deformation, progressive alignment of grains with increasing foliation intensity (Figure 3a), and structures indicating local dissolution and precipitation (Figures 3b and 3e), bulk strain and associated foliation development is inferred to be accomplished by a combination of dissolution‐precipitation creep, grain rotation and face‐on‐face sliding between (001) facets (Figure 5). Face‐on‐face sliding is commonly suggested to be an important deformation mechanism in phyllosilicate‐rich rocks (e.g., Bos & Spiers, 2002; Hansen et al., 2020; Niemeijer, 2018; Seguí et al., 2021), as is dissolution‐precipitation creep under fluid‐present conditions (e.g., Bos & Spiers, 2002; Niemeijer, 2018; Wenk et al., 2019; Wintsch & Yi, 2002). Structures developed in lizardite‐chrysotile serpentinite deformed in oceanic transforms (Cox et al., 2021), and the San Andreas fault system (Andreani et al., 2005) also indicate a similar deformation mechanisms.

## 6 Rheology of Antigorite

Phyllosilicate‐rich rocks are substantially weaker when sliding occurs at low angles to aligned (001) facets (e.g., Collettini et al., 2009; Niemeijer, 2018). Consequently, alignment of antigorite (001) facets during foliation development should allow more effective face‐on‐face sliding. Although antigorite grains have a large aspect ratio, such that grain size in thin section is dependent on orientation, the length of the smallest axis decreases with increasing foliation intensity, suggesting that grain size reduction accompanies foliation development. Accordingly, dissolution‐precipitation creep should become more effective (Bos & Spiers, 2002; Paterson, 1995; Rutter, 1976) as the foliation develops. Therefore, we expect that foliation development during progressive shear drives progressive weakening (Figure 5), consistent with that poorly foliated antigorite forms competent lenses in a well‐foliated matrix. Once the foliation is developed, the strain‐dependence should diminish, and strength should be controlled by the combined operation of dissolution‐precipitation creep and face‐on‐face sliding between (001) facets (Figure 5c).

At conditions where dissolution‐precipitation creep is less efficient than other grain‐scale mechanisms, such as, but not limited to, at higher strain rate (and higher differential stress), cooler temperatures or larger grain size, we expect increased activity of other deformation mechanisms. For example, at strain rates employed in laboratory creep experiments (∼10^−5^ s^−1^), that are greater than inferred for the NMR (∼10^−12^ s^−1^; Tulley et al., 2020), deformation commonly results in localized shear zones showing cataclastic textures (Chernak & Hirth, 2010; French et al., 2019; Gasc et al., 2017; Hirauchi et al., 2020; Proctor & Hirth, 2016), that are uncommon in naturally deformed antigorite (Figure 3a; Hirauchi et al., 2021; Padrón‐Navarta et al., 2012; Wassmann et al., 2011). This is consistent with an increase in the activity of frictional mechanisms toward higher strain rates. Frictional mechanisms (including face‐on‐face sliding) should also be more active where effective stresses are low (Bos & Spiers, 2002; Hirauchi et al., 2021), for example, in regions of high fluid pressure along subduction plate boundaries. Variations in the relative contributions of frictional and viscous mechanisms are also consistent with the suggestion that antigorite may host earthquake slip at higher strain rates (Wang et al., 2020).

## 7 Conclusion

Outcrops of the NMR at Mie show antigorite serpentinite deformed at 474°C ± 30°C within a broad viscous shear zone that experienced a strain rate ∼10^−12^ s^−1^. Our observations indicate that the dominant deformation mechanisms in this natural shear zone are dissolution‐precipitation creep and face‐on‐face sliding between (001) crystal facets, giving a frictional‐viscous rheology. At conditions where dissolution‐precipitation creep is less effective, such as at higher natural strain rates or in the laboratory, or under fluid‐absent conditions, we expect that frictional behavior and dislocation glide will become more dominant, leading to strain localization.

## Supporting information

Supporting Information S1

## Data Availability

EBSD data and matlab code used for analysis are available from the Cardiff University Research Portal (http://doi.org/10.17035/d.2022.0164034284).
