# Supplementary material for: Rheology of Naturally Deformed Antigorite Serpentinite: Strain and Strain‐Rate Dependence at Mantle‐Wedge Conditions
Source: Geophys Res Lett. 2022 Aug 26;49(16):e2022GL098945. doi: 10.1029/2022GL098945 (PMC9539589; doi:10.1029/2022GL098945)
Supplement: Supplementary file 1 — Supporting Information S1 [file GRL-49-e2022GL098945-s001.pdf]

Supporting Information for "Rheology of antigorite at mantle-wedge conditions - strain and strain-rate dependence"

C. J. Tulley[1], Å. Fagereng[1], K. Ujiie[2], S. Piazolo[3], M. S. Tarling[4] *, Y. Mori[5]

[1]School of Earth and Environmental Sciences, Cardiff University, Cardiff, Wales, United Kingdom.

[2]Faculty of Life and Environmental Sciences, University of Tsukuba, Tsukuba, Japan.

[3]School of Earth and Environment, University of Leeds, Leeds, United Kingdom.

[4]Department of Geology, University of Otago, Dunedin, New Zealand.

[5]Kitakyushu Museum of Natural History and Human History, Kitakyushu, Japan.

Contents of this file

1. Text S1
2. Figure S1

Corresponding author: C. J. Tulley, School of Earth and Environmental Sciences, Cardiff University, Cardiff, Wales, United Kingdom. (tulleycj@gmail.com)

*Now at Department of Earth and Planetary Sciences, McGill University

Introduction Figure S1 shows representative Raman spectra obtained from non-foliated and foliated serpentinite, and a serpentinite vein within the Mie serpentinite. Text S1 describes the features of these spectra, and outlines the interpretation of the serpentine polytype.

Text S1. Poorly- and intensely-foliated serpentine, and serpentine veins, show similar Raman spectra (Fig. S1a). The shape of the spectra does not vary between fabric types and is similar to the shape of previously analysed antigorite spectra (Fig. S1b); although, compared to published antigorite spectra and reference antigorite samples analysed during the same analytical session, these spectra lack a prominent trough near 3687 cm^{-1}.

In the analysed samples, the strongest peak is consistently centred near 3663 cm^{-1} (Fig. S1a). Two smaller peaks with similar intensities appear near 3683 cm^{-1}, and 3695 cm^{-1}. The strongest peak in the studied samples, near 3663 cm^{-1}, occurs at slightly lower wavenumber than the strongest peak in previously analysed antigorite samples, which occurs between 3665-3670 cm^{-1}. The peak near 3695 cm^{-1} is within the range of positions between 3695-3700 cm^{-1} reported for the smaller antigorite peak in the high wavenumber region. The peak near 3683 cm^{-1} is close to a lizardite peak which occurs in the range 3683-3684 cm^{-1} (Rooney et al., 2018); however, a shoulder near 3701-3703 cm^{-1} is present in lizardite spectra but not in spectra from Mie serpentinite. None of the observed spectra show shapes or peak positions consistent with chrysotile.

Electron images of serpentine grains (Figs. 3d–3f in main text) show no variation in mineral habit which might reflect intergrowth of different minerals or serpentine polytypes,

so this seems an unlikely explanation for the unusual spectra shape. We infer that the Mie serpentine is the antigorite polytype (as also suggested by Hirauchi et al., 2020), based on Raman spectra in the low-wavenumber region), but has a slightly different chemical structure than previously analysed antigorite. Overall, Raman spectra suggest that the Mie serpentinite is the antigorite polytype, and imply that there is no variation in polytype between the fabric types.

References

Hirauchi, K.-i., Katayama, I., & Kouketsu, Y. (2020). Semi-brittle deformation of antigorite serpentinite under forearc mantle wedge conditions. *Journal of Structural Geology*, *140*, 1–10. doi: 10.1016/j.jsg.2020.104151

Petriglieri, J. R., Salvioli-Mariani, E., Mantovani, L., Tribaudino, M., Lottici, P. P., Laporte-Magoni, C., & Bersani, D. (2015). Micro-Raman mapping of the polymorphs of serpentine. *Journal of Raman Spectroscopy*, *46*(10), 953–958. doi: 10.1002/jrs.4695

Rooney, J. S., Tarling, M. S., Smith, S. A. F., & Gordon, K. C. (2018). Submicron Raman spectroscopy mapping of serpentinite fault rocks. *Journal of Raman Spectroscopy*, *49*, 279–286. doi: 10.1002/jrs.5277

Tarling, M. S., Smith, S. A. F., Scott, J. M., Rooney, J. S., Viti, C., & Gordon, K. C. (2019). The internal structure and composition of a plate-boundary-scale serpentinite shear zone: the Livingstone Fault, New Zealand. *Solid Earth*, *10*(4), 1025–1047. doi: 10.5194/se-10-1025-2019

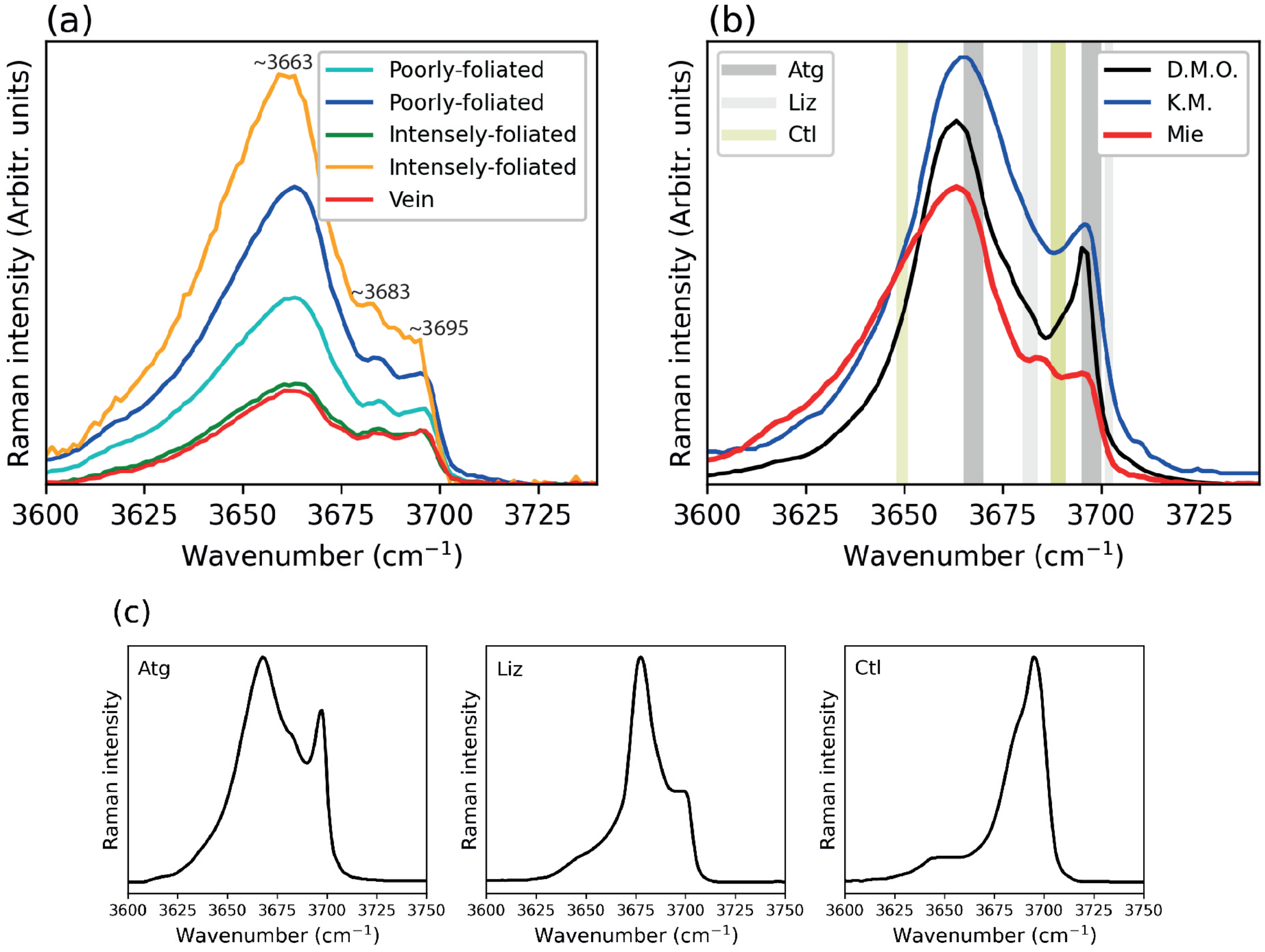

Figure S1. Raman spectra from Mie serpentinite and reference antigorite samples. (a) Spectra and peak positions for poorly- and intensely-foliated, and vein serpentinite from the study area. (b) Comparison between a representative spectrum from Mie serpentinite, a spectrum obtained from an antigorite reference sample from the Dun Mountain Ophiolite (D.M.O.) (Tarling et al., 2019), and the antigorite spectrum presented in Petriglieri et al. (2015), from the Koniambo Massif (K.M). Vertical lines show the range of peak positions previously reported for antigorite (Atg), lizardite (Liz), and chrysotile (Ctl), based on the compilation in Rooney et al. (2018). (c) Typical shapes of lizardite, chrysotile, and antigorite spectra, modified from Rooney et al. (2018).
